# Media Use and Its Associations With Paranoia in Schizophrenia and Bipolar Disorder: Ecological Momentary Assessment

**DOI:** 10.2196/59198

**Published:** 2024-07-03

**Authors:** Vincent Paquin, Robert A Ackerman, Colin A Depp, Raeanne C Moore, Philip D Harvey, Amy E Pinkham

**Affiliations:** 1 Department of Psychiatry, McGill University, Montreal, QC, Canada; 2 Lady Davis Institute for Medical Research, Jewish General Hospital, Montreal, QC, Canada; 3 Douglas Mental Health University Institute, Montreal, QC, Canada; 4 School of Behavioral and Brain Sciences, The University of Texas at Dallas, Richardson, TX, United States; 5 Department of Psychiatry, University of California San Diego, La Jolla, CA, United States; 6 Department of Psychiatry and Behavioral Sciences, University of Miami, Miami, FL, United States

**Keywords:** paranoia, social media, digital media, technology, psychosis, schizophrenia, schizoaffective, bipolar disorder, ecological momentary assessment, spectrum, sociodemographic, linear mixed model, media use, mental health, digital intervention, adult, adults, medical center, mental health clinic, psychiatry, psychiatrist

## Abstract

**Background:**

Paranoia is a spectrum of fear-related experiences that spans diagnostic categories and is influenced by social and cognitive factors. The extent to which social media and other types of media use are associated with paranoia remains unclear.

**Objective:**

We aimed to examine associations between media use and paranoia at the within- and between-person levels.

**Methods:**

Participants were 409 individuals diagnosed with schizophrenia spectrum or bipolar disorder. Measures included sociodemographic and clinical characteristics at baseline, followed by ecological momentary assessments (EMAs) collected 3 times daily over 30 days. EMA evaluated paranoia and 5 types of media use: social media, television, music, reading or writing, and other internet or computer use. Generalized linear mixed models were used to examine paranoia as a function of each type of media use and vice versa at the within- and between-person levels.

**Results:**

Of the 409 participants, the following subgroups reported at least 1 instance of media use: 261 (63.8%) for using social media, 385 (94.1%) for watching TV, 292 (71.4%) for listening to music, 191 (46.7%) for reading or writing, and 280 (68.5%) for other internet or computer use. Gender, ethnoracial groups, educational attainment, and diagnosis of schizophrenia versus bipolar disorder were differentially associated with the likelihood of media use. There was a within-person association between social media use and paranoia: using social media was associated with a subsequent decrease of 5.5% (fold-change 0.945, 95% CI 0.904-0.987) in paranoia. The reverse association, from paranoia to subsequent changes in social media use, was not statistically significant. Other types of media use were not significantly associated with paranoia.

**Conclusions:**

This study shows that social media use was associated with a modest decrease in paranoia, perhaps reflecting the clinical benefits of social connection. However, structural disadvantage and individual factors may hamper the accessibility of media activities, and the mental health correlates of media use may further vary as a function of contents and contexts of use.

## Introduction

### Background

Paranoia, defined as the “unfounded fear that others intend to cause you harm” [1], is the most commonly identified delusion in schizophrenia spectrum conditions [2] and is also experienced in bipolar disorder, unipolar depression, borderline personality disorder, and other conditions [3-5]. Paranoia is a spectrum of beliefs and experiences spanning mild (eg, people talk about you behind your back) to severe ideas of threat (eg, people are trying to harm you) [6]. Paranoid experiences along this hierarchy are associated with social functioning problems including poorer interpersonal relationships, reduced marital satisfaction, and difficulties with peers [7-12]. Paranoia can fluctuate over the course of days as a function of cognitive and interpersonal factors [13-15]. Factors that have been associated with increased paranoia include rumination, loneliness, and social exclusion, whereas distraction and being in the company of familiar individuals have been associated with decreases in paranoia [13,14,16,17]. Many of these social and cognitive experiences may be influenced by day-to-day engagement with media [18], but it is unclear whether media use influences paranoia in schizophrenia and bipolar disorder.

### Possible Effects of Media on Paranoia

Media are vehicles for accessing or sharing information, including for leisure, social, and occupational purposes [19]. This definition encompasses both digital (eg, social media) and traditional (eg, books) forms of media. Similar to other environmental exposures, media may potentially influence a person’s paranoia by informing the degree of perceived environmental threat. Better situating the naturalistic effects of media on paranoia may help guide lifestyle counseling and the development of media-based interventions for this symptom dimension.

Multiple mechanisms might contribute to the effects of media on paranoia. First, media activities can provide a distraction that helps a person move away from persecutory thoughts. Social media may help decrease paranoia by facilitating access to social support and reducing loneliness. However, both traditional and digital media activities may also promote paranoia if they perpetuate harmful avoidance behaviors (eg, if a socially withdrawn person watches TV instead of going out and seeing friends) or directly amplify the perception of environmental threat (eg, through consumption of catastrophic news or conspiracist discourses). Additionally, social media has several features that distinguish it from in-person communication; some of these features, such as asynchronicity and lower availability of nonverbal cues, may hypothetically increase uncertainty about social relationships [20], which might build on a person’s paranoia.

There is currently limited evidence regarding the relationship between media use and paranoia. Recent studies with the general population (in Canada and the United Kingdom) have found cross-sectional associations between greater use of digital media and higher levels of psychotic-like experiences [21,22], including paranoia [23], but 2 studies with prospective data did not find robust associations between media use and the subsequent risk of psychotic-like experiences [22,24]. Rather than reflecting causal effects of media on paranoia, the concurrent association between media use and paranoia in the general population may be due to confounders, such as preexisting mental health problems and social adversity [24]. However, these findings may not apply to individuals with psychotic or mood disorders, in whom there is generally a greater propensity for paranoia, and thus potentially a greater sensitivity to media effects that are otherwise not apparent in non-clinical populations. As such, surveys indicate that 25%‐35% of individuals with psychotic or mood disorders believe that digital devices can increase their paranoia [25,26].

To our knowledge, only 1 previous study has examined the associations of media use and paranoia in a clinical sample [27]. This study by Berry et al [27] included 44 participants: 19 individuals with schizophrenia spectrum or bipolar disorder and 25 nonclinical individuals. Participants completed ecological momentary assessments (EMAs) of social media use and paranoia 6 times daily over 6 days. Posting about feelings, venting on social media, viewing profiles of people who were not “friends,” and lower perceived social rank were associated with higher paranoia at the next time point. The results therefore supported a possible contribution of specific social media experiences to paranoia. However, the results may have been affected by unmeasured confounders, such as individual differences in social adversity or mental health that predisposed individuals to certain patterns of social media behaviors. In addition, because the usage of other types of media was not assessed, it remains unclear to what extent the risk is specific to social media. Another potential gap is whether day-to-day fluctuations in paranoia reciprocally make people more or less drawn to media use [28]—a reverse association that has yet to be examined.

### Population Differences in Media Use and Paranoia

An important consideration in investigating the relationship of media activities with paranoia is that media-related behaviors vary between populations. Some studies have found, for example, that people with schizophrenia are less likely than those with bipolar disorder to use digital media [29,30], perhaps reflecting the impact of socioeconomic disadvantage on media access and digital literacy. Similarly, in the general population, there is evidence of unequal access to media technologies as a result of socioeconomic disadvantage [31,32]. Gender is another factor linked to differences in media use, with meta-analytic evidence of higher prevalence of problematic social media use in women versus men [33].

The risk of paranoia may also vary between populations. Although there is limited comparative data, paranoia appears to be common in both schizophrenia and mood disorders and to have similar psychosocial and neurobiological correlates across diagnostic groups [2,4,34,35]. One study found no difference in patterns of attributional bias between schizophrenia and bipolar disorder, suggesting shared cognitive mechanisms [34]. However, paranoia appears to be relatively less common in unipolar depression with psychotic features than in schizophrenia and bipolar disorder, indicating different levels of risk between diagnoses [36]. Likewise, in the general population, levels of paranoia may be higher among men than among women [8]. Whether these epidemiological features might translate into diagnosis- or gender-specific effects of media on paranoia remains unknown.

### This Study

To advance knowledge about the relationship between media use and paranoia, this study draws from an EMA sample comprising adults with schizophrenia and those with bipolar disorder. EMA provides dense, repeated measures that are well suited for analyzing bidirectional relationships between media use and paranoia. With EMA data, confounders from individual traits can be removed using within-person analyses, where each person is their own comparator over time [37]. We thus aimed to examine media use and its bidirectional associations with paranoia in people with schizophrenia and those with bipolar disorder, both at the between- and within-person levels. To identify whether specific types of media activities are uniquely associated with paranoia, we considered social media and 4 other types of media use: watching TV, listening to music, reading or writing, and internet or computer use. We first evaluated associations of sociodemographic and clinical characteristics with any versus no use of each type of media across the study period. Then, we examined the associations between media use and paranoia at the between- and within-person levels. We explored moderating effects of gender and clinical group on within-person associations. We hypothesized that social media use would be associated with subsequent increases in paranoia, and that this association would be greater in women than in men. We did not formulate hypotheses for other types of media.

## Methods

### Participants

Data were obtained from a study designed to assess the introspective accuracy of self-reported cognition and functioning in the context of mental illness [38]. Participants were adults aged 18‐65 years meeting diagnostic criteria for schizophrenia (or schizoaffective disorder) and bipolar disorder (type 1 or 2). Recruitment sites included The University of Texas at Dallas, Miller School of Medicine–University of Miami, and University of California San Diego. Participants were recruited via medical centers, public mental health and local community clinics, nonprofit organizations, direct contact by service providers, follow-up with research participants from previous projects, study flyers, and internet-based advertisements.

Diagnostic information was collected by trained interviewers using the Mini International Neuropsychiatric Interview [39] and the psychosis module of the Structured Clinical Interview for *DSM-5* (*Diagnostic and Statistical Manual of Mental Disorders* [Fifth Edition]) [40]. Final diagnoses were generated through a local consensus procedure. Inclusion criteria were clinical stability (ie, no hospitalization or extended emergency department visit) for a minimum of 6 weeks and no significant (>20%) medication dose changes in the past 2 weeks. Participants with bipolar disorder had to have at least 1 mood episode recurrence or incomplete remission from a first episode, indicating stage 3 severity or higher according to the classification of Frank et al [41]. Exclusion criteria were nonproficiency in English; a current or past medical or neurological disorder that may affect brain functioning (eg, brain tumors and seizures); intellectual disability or pervasive developmental disorder; active substance use of moderate severity or higher; and any visual or hearing impairment limiting assessments.

### Baseline Assessments

During the initial interview, participants reported their age, gender (male, female, or other), race (Asian, Black or African American, White, or other), ethnicity (Hispanic or non-Hispanic), educational attainment, and relationship status. Trained raters assessed symptom severity the day before the first EMA survey using the Positive and Negative Syndrome Scale (PANSS) [42] for schizophrenia-related symptoms, the Montgomery-Åsberg Depression Rating Scale for depressive symptoms [43], and the Young Mania Rating Scale [44] for mania-related symptoms.

The PANSS consists of 30 items, each scored on a scale from 1=“Absent” to 7=“Extreme.” Its positive symptom subscale (7 items, total score range 7‐49) includes 1 item that measures the severity of suspiciousness and paranoia (P6). Negative symptoms were assessed with the PANSS using the 2-factor model by developed Khan et al [45], which identifies dimensions of expressive and experiential deficits. The items for reduced emotional experience include emotional withdrawal (N2), passive or apathetic social withdrawal (N4), and active social avoidance (G16). The items for reduced emotional expression include blunted affect (N1), poor rapport (N3), lack of spontaneity and flow of conversation (N6), and motor retardation (G7).

### EMAs

Participants completed EMA surveys via the NeuroUX platform (Playpower Labs Inc) with either their own smartphone or a smartphone provided by the study investigators. The initial protocol planned that all participants would be provided smartphones, but participants were allowed to use their own smartphones following the onset of the COVID-19 pandemic to facilitate more flexible participation. In the context of this rapid modification of the protocol, data on smartphone ownership were not collected systematically and thus could not be analyzed.

Participants received SMS text messages prompting them to complete internet-based surveys 3 times daily for 30 days. Data were instantly uploaded to cloud-based servers. Text messages were sent at stratified random intervals within, on average, 2-hour windows and between 9 AM and 9 PM. The first and last daily assessment times were adapted to each participant’s typical sleep and wake schedules. Survey responses were only allowed within 1-hour periods following deployment of the SMS text messages. Participants had the option of silencing the alarms for 30-minute intervals (eg, if driving).

Media use was measured using checkbox questions about activities performed since the previous survey. Response options included six items: (1) using “social media (eg, Facebook, Twitter)”; (2) “watching TV”; (3) “listening to music”; (4) “reading, writing, or journaling”; (5) “shopping online”; and (6) “other internet, computer, or tablet use.” Due to low endorsement rates for shopping on the web, this activity was merged with other internet, computer, or tablet uses. Other types of activities, such as working, excising, eating, etc, were also measured as part of this questionnaire [46] but were not analyzed in this study. Participants could report multiple activities per assessment. For each type of media, these checkbox questions produced dichotomous indices of use at each survey (0=did not use and 1=used).

Paranoia was measured using the following item: “Since the past alarm, how much have you had thoughts that others might want to harm you or that people are untrustworthy?” Response choices ranged from 1 to 7, with higher values indicating greater severity or frequency. In support of the convergent validity of this item, we found that individual mean scores for paranoia across the 30 days were significantly correlated with scores on the PANSS suspiciousness item (Spearman ρ=0.41, *P*<.001). Both media use and paranoia were measured 3 times daily.

### Statistical Analysis

Analyses were conducted in R (version 4.1.2; The R Foundation). Codes are available on the web [47]. To minimize the impact of potentially invalid entries, we excluded participants who completed less than one-third of EMA surveys, following a common rule of thumb in EMA research [48]. Characteristics of included and excluded participants were compared to consider attrition effects; in line with recommendations for descriptive statistics [49], we used effect sizes instead of *P* values for these comparisons. Descriptive analyses such as the root-mean-square of successive differences (RMSSD) and the intraclass correlation coefficient were used to evaluate the variability of paranoia EMAs [50].

To examine sociodemographic and clinical correlates of media use, we dichotomized each type of media use as any versus no use over the follow-up period. We regressed these dichotomous variables on the predictors using logistic regressions adjusted for age and gender. To account for pairwise evaluations of 16 predictors with each media type, we report nominal statistical significance after adjustment for the false discovery rate (using the Benjamini and Hochberg [51] method) in addition to uncorrected 95% CIs.

Next, to examine associations between ecological momentary reports of media use and paranoia (between and within persons), we used generalized mixed models with observations nested in individuals [52]. Models were estimated using maximum likelihood with Laplace approximation. We considered 2 directions of association: media use as a function of paranoia, and paranoia as a function of media use. For models of media use as a function of paranoia, we applied the binomial distribution (logistic regression) given that media use assessments were dichotomous. For models of paranoia as a function of media use, we applied a gamma distribution with log-link function given the skewed distribution of paranoia. All models included random intercepts, mean levels of the predictor, and lagged mean-centered values of the predictor. Random slopes of the lagged mean-centered predictor were also added if they improved model fit (*P*<.05) on the likelihood ratio test [53]. Values of 95% CI not overlapping the null were considered significant.

For each model of media use and paranoia, we subsequently explored potential moderating effects of gender (0=male, 1=female) and clinical group (0=schizophrenia, 1=bipolar) on within-person associations. Interactions were considered statistically significant at *P*<.05. Recognizing, however, that interaction analyses are frequently underpowered due to weaker effects [54], we also probed interactions if the *P* value ranged between .05 and .10 and reported them as “tentative.” To probe interactions, we estimated marginal slopes of lagged mean-centered predictors as a function of the moderator [55].

In sensitivity analyses, we considered whether including all participants (ie, not removing participants who completed less than one-third of EMA surveys) impacted the primary results. We also considered the impact of auto-correlation by controlling for the lagged dependent variables.

### Ethical Considerations

All participants provided written informed consent, and the institutional review board of each university approved the study (The University of Texas at Dallas, #18‐93; Miller School of Medicine–University of Miami, #20180352; and University of California San Diego, #180716).

## Results

Of 446 participants, 409 completed at least one-third of the EMA surveys (Table 1). Completion of at least one-third of assessments was associated with the site of recruitment, higher educational attainment, and non-Hispanic ethnicity (effect sizes≥0.100; see Table S1 in Multimedia Appendix 1). Table 2 provides descriptive statistics on paranoia EMAs, indicating higher mean ratings and greater intraindividual variability (higher SD and RMSSD) of paranoia in the schizophrenia versus bipolar disorder group.

**Table 1.** Characteristics of participants in the schizophrenia and bipolar disorder groups. Positive symptoms were measured with the positive symptom subscale of the Positive and Negative Syndrome Scale (PANSS; score range 7-49). Reduced emotional experience (range 3-21) and reduced emotional expression (range 4-28) were measured with negative symptom and general psychopathology items of the PANSS. Depressive symptoms were measured with the Montgomery-Åsberg Depression Rating Scale (range 0-60). Mania-related symptoms were measured with the Young Mania Rating Scale (range 0-60).

		Schizophrenia (n=189)	Bipolar disorder (n=220)
**Site of recruitment, n (%)**			
	The University of Texas at Dallas	86 (45.5)	87 (39.5)
	University of Miami	61 (32.3)	54 (24.5)
	University of California San Diego	42 (22.2)	79 (35.9)
Age (years), median (IQR)		41 (32-52)	39 (30-50)
**Gender, n (%)**			
	Male	93 (49.2)	69 (31.4)
	Female	95 (50.3)	150 (68.2)
	Other	<5 (<1)	<5 (<1)
**Race, n (%)**			
	White	72 (38.1)	140 (63.6)
	Black or African American	96 (50.8)	42 (19.1)
	Asian	5 (2.65)	15 (6.82)
	Other	16 (8.47)	23 (10.5)
**Ethnicity, n (%)**			
	Hispanic	40 (21.2)	56 (25.6)
	Non-Hispanic	149 (78.8)	163 (74.4)
**Educational level, n (%)**			
	High school diploma or less	83 (43.9)	40 (18.2)
	Some college	71 (37.6)	77 (35.0)
	College degree or higher	35 (18.5)	103 (46.8)
**Relationship status, n (%)**			
	Not in a relationship	115 (60.8)	101 (45.9)
	In a relationship	74 (39.2)	119 (54.1)
Positive symptoms, median (IQR)		16 (13-19)	11 (9-14)
Reduced emotional experience, median (IQR)		6 (3-8)	3 (3-6)
Reduced emotional expression, median (IQR)		6 (4-9)	4 (4-6)
Depressive symptoms, median (IQR)		6 (0-16)	11 (0-20)
Mania-related symptoms, median (IQR)		0 (0-0)	0 (0-5)

**Table 2.** Descriptive statistics of paranoia ecological momentary assessments. Person-level statistics were calculated for each participant then averaged (with SD values) across individuals. *P* values indicate group differences in these statistics based on *t* tests. The intraclass correlation coefficient (ICC) corresponds to the proportion of between-person variance; the proportion of within-person variance is 1–ICC.

Descriptor		Diagnostic group		*P* value
		Schizophrenia (n=189)	Bipolar disorder (n=220)	
**Number of surveys across participants, n**				
	No paranoia (score=1)	6685	12,630	—^a^
	Some paranoia (score>1)	6445	2684	—
	Missing	3880	4486	—
	Total	17,010	19,800	—
Some paranoia (score>1) on at least 1 assessment, n (%)		164 (86.6)	123 (55.9)	—
Person-level mean of paranoia, mean (SD)		2.45 (1.57)	1.57 (1.18)	<.001
Person-level SD of paranoia, mean (SD)		0.91 (0.65)	0.47 (0.66)	<.001
Person-level RMSSD^b^ of paranoia, mean (SD)		0.93 (0.69)	0.48 (0.68)	<.001
ICC^c^ (95% CI)		0.66 (0.61-0.71)	0.69 (0.65-0.73)	—

a Not applicable.

b RMSSD: root-mean-square of successive differences.

c ICC: intraclass correlation coefficient.

Of the 409 participants, the following subgroups reported at least 1 instance of media use: 261 (63.8%) for using social media, 385 (94.1%) for watching TV, 292 (71.4%) for listening to music, 191 (46.7%) for reading or writing, and 280 (68.5%) for other internet or computer use. Within each of these subgroups, there was a total of 1982 reports of social media over 18,478 available surveys (10.7%), 6779 reports of watching TV over 26,911 available surveys (25.2%), 1997 reports of listening to music over 20,778 available surveys (9.6%), 864 reports of reading or writing over 13,478 available surveys (6.4%), and 2500 reports of other internet or computer use over 19,649 available surveys (12.7%).

### Factors Associated With Any Versus No Media Use

**Figure 1.**
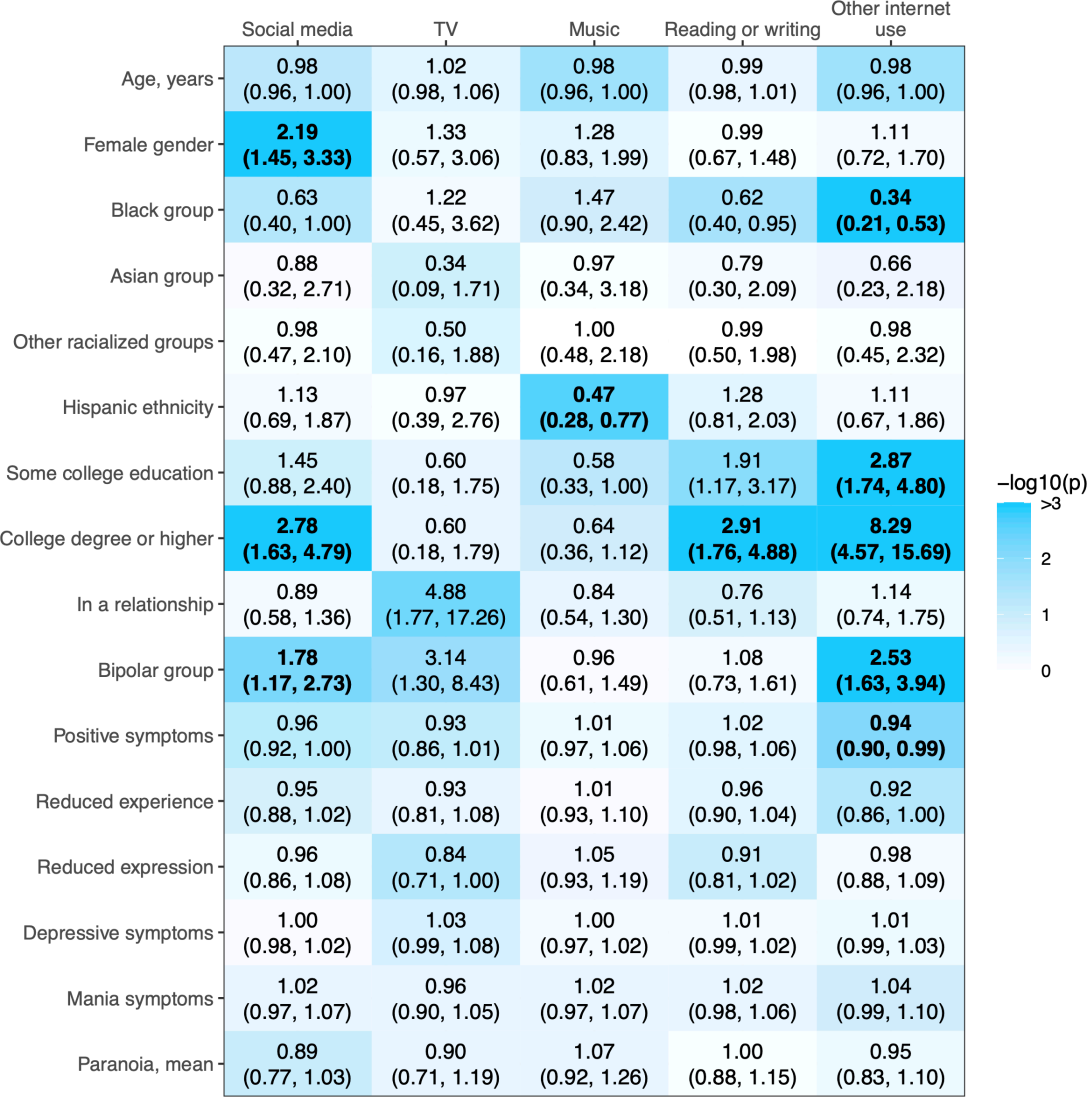
Odds ratios (95% CIs) of any versus no use of each type of media as a function of participant characteristics. Logistic regression models were adjusted for age and gender (n=409). Statistical significance after adjustment for the false discovery rate across columns (adjusted *P*<.05) is indicated in boldface. Comparator category for female gender: male gender; for Black or African American (“Black”), Asian, and other racialized groups: White; for Hispanic ethnicity: non-Hispanic ethnicity; for some college education and college degree or higher: high school or lower; and for the bipolar group: the schizophrenia group. Paranoia is the mean rating for paranoia across ecological momentary assessments (range 1‐7).

Figure 1 presents factors associated with any versus no use of each type of media. After adjustment for the false discovery rate, female versus male gender was associated with higher odds of social media use. Black or African American individuals (versus White individuals) had lower odds of other internet or computer use. Hispanic versus non-Hispanic ethnicity was associated with lower odds of listening to music. Higher educational attainment was associated with higher odds of social media use, reading or writing, and other internet or computer use. Diagnosis of bipolar disorder versus schizophrenia was associated with higher odds of social media and other internet or computer use. Higher levels of positive symptoms (but not paranoia) were associated with lower odds of other internet or computer use.

### Paranoia as a Function of Media Use

**Figure 2.**
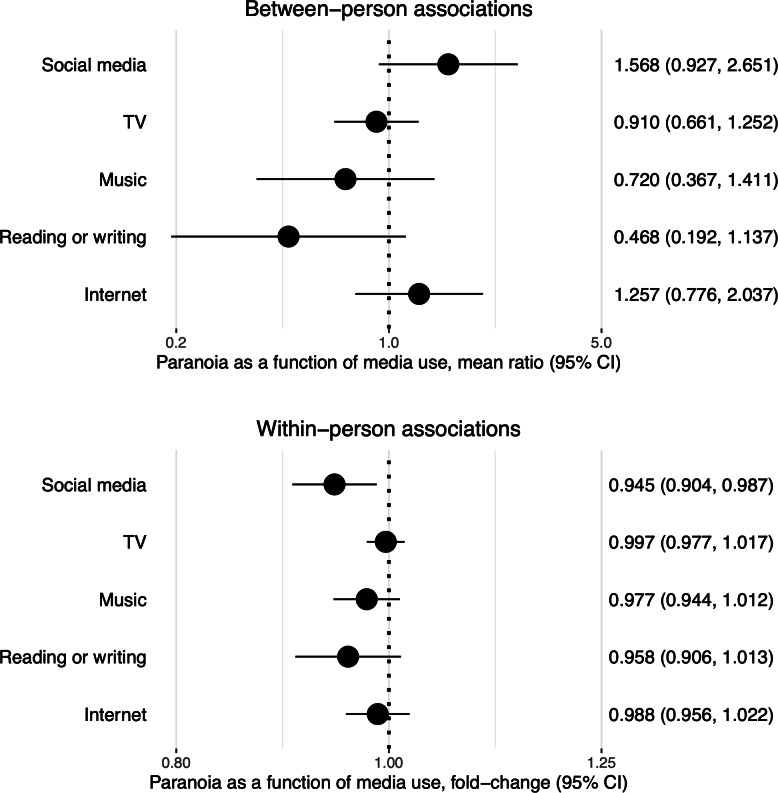
Paranoia as a function of preceding (lagged) media use in gamma mixed models with observations nested within individuals. Predictors included mean levels of media use (for between-person associations) and lagged mean-centered media use (for within-person associations). All models include random slopes of lagged mean-centered media use.

Figure 2 presents the mean ratio (between persons) and fold-change (within persons) in paranoia as a function of media use. Analyses are in the subsets of participants reporting that type of media (ie, subgroups ranging from n=191 for reading or writing to n=385 for watching TV). Across types of media, associations were not significant at the between-person level: participants’ average levels of media use were not associated with their average levels of paranoia. At the within-person level, social media use above a person’s average was associated with a ~5.5% reduction in their level of paranoia on the subsequent survey (fold-change 0.945; 95% CI 0.904-0.987). Other within-person associations were not significant.

There was a tentative moderating effect of the clinical group on the within-person association of social media use with paranoia (coefficient, lagged social media use×bipolar disorder versus schizophrenia=1.084; *P*=.07). According to this interaction, there was a within-person association between social media use and subsequently reduced paranoia in the schizophrenia group (fold-change 0.900, 95% CI 0.840-0.965). In the bipolar disorder group, however, the same association was not statistically significant (fold-change 0.975, 95% CI 0.923-1.031). There was also a tentative moderating effect of the clinical group on the association between listening to music and paranoia (coefficient, lagged music×bipolar disorder versus schizophrenia=0.939; *P*=.08). According to this interaction, there was a within-person association between listening to music and subsequently decreased paranoia in the bipolar disorder group (fold-change 0.949, 95% CI 0.906-0.995). This association was not significant in the schizophrenia group (fold-change 1.011, 95% CI 0.961-1.063). Other moderating effects of gender or clinical group did not pass the *P*<.10 threshold and were not probed further.

### Media Use as a Function of Paranoia

**Figure 3.**
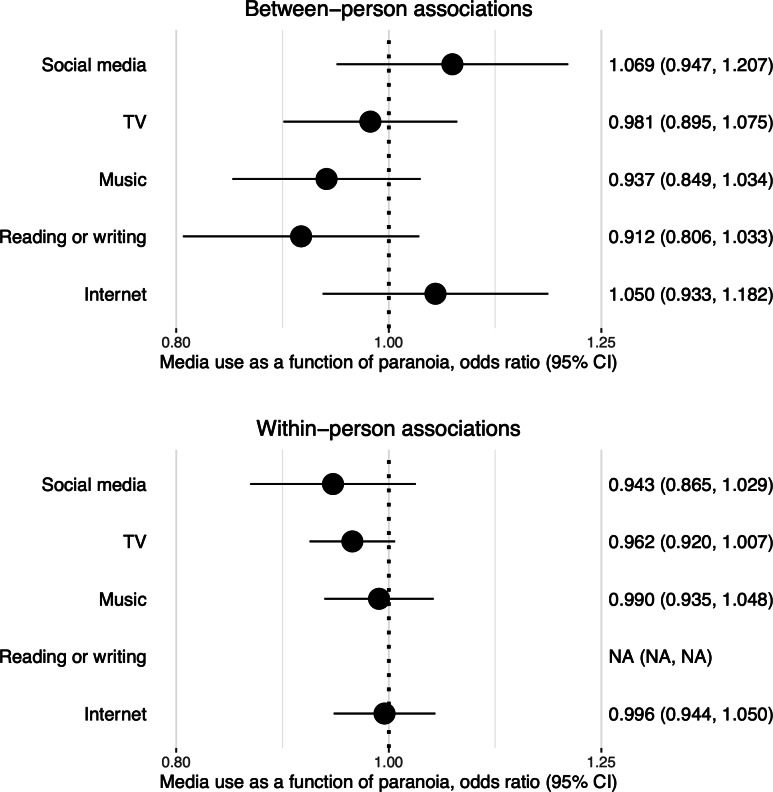
Media use as a function of preceding (lagged) paranoia in generalized logistic mixed models with observations nested in individuals. Predictors include mean levels of paranoia (for between-person associations) and lagged mean-centered paranoia (for within-person associations). Models of social media use and TV include random slopes of lagged mean-centered paranoia. Other models include random intercepts only. Lagged mean-centered paranoia was removed from models of reading or writing due to lack of convergence (indicated by “NA” in the bottom panel).

Figure 3 presents odds ratios of each type of media use as a function of paranoia. Associations were not significant at the between-person level: participants’ average levels of paranoia were not associated with the odds of media use. There were also no significant associations at the within-person level: participants’ variations in paranoia relative to their personal average did not predict variations in their odds of media use over time. The moderating effects of gender or clinical group over within-person associations did not pass the threshold of *P*<.10 and were not probed further.

### Additional Analyses

Within- and between-person associations of media use and paranoia were consistent in the full sample, that is, after lifting the exclusion rule for participants with less than one-third of EMA surveys (see Figures S1 and S2 in Multimedia Appendix 1). Results were also consistent in models adjusted for lagged values of the dependent variables, suggesting no impact of auto-correlated effects on the association between lagged social media use and lower paranoia (see Figures S3 and S4 in Multimedia Appendix 1). Post hoc, we explored associations between media use and paranoia on concurrent surveys, instead of across lagged surveys. None of the concurrent associations were significant (see Figures S5 and S6 in Multimedia Appendix 1).

## Discussion

### Principal Findings

In a large cohort of individuals with schizophrenia or bipolar disorder, we examined the usage of social media and 4 other types of media, and their associations with paranoia over 30 days of EMA surveys. Sociodemographic and clinical characteristics were associated with the likelihood of any versus no use of media types. In individuals who did use those media over the follow-up period, media use was either associated with no significant change in paranoia, or some decrease, depending on the type of media and diagnostic group.

We did not find evidence that media use predicted increased paranoia overall, or that paranoia predicted subsequent media use. Contrary to our hypothesis, there was instead an association between social media use and a subsequent decrease in paranoia. The reduction was approximately 5.5% in the total sample and up to 10% in the schizophrenia group. Although there are no established benchmarks for interpreting the clinical significance of paranoia reductions on the present scale, it should be noted that these reductions are smaller than 1-point changes relative to the 7-point range of the scale, leading us to interpret them as modest. The reverse associations (between paranoia and subsequent social media use) and concurrent associations (between paranoia and concurrent social media use) were not significant, supporting a temporal sequence between social media use and subsequent decreases in paranoia.

Although social media may have negative effects on mental health through negative social comparisons, increased uncertainty in social communication, and other mechanisms [20,27,56], there is evidence that people with psychosis [57] and other mental health conditions [58] primarily use social media to connect with friends or family. Other studies have found that (physical) exposure to familiar social company and lower levels of loneliness are associated with decreased paranoia [14,16]. Building on this literature, this finding may reflect the effect of web-based social company through social media on reducing paranoia. The fact that this association was stronger in the schizophrenia versus bipolar disorder group is of unclear significance. In the literature, loneliness has been reported as a risk factor for paranoia regardless of whether people have a psychotic disorder [16], suggesting that increased social company through social media could hypothetically be protective across diagnostic groups. We believe that the lack of an association between social media and paranoia in the bipolar disorder group may be a consequence of the group’s lower intraindividual variation in paranoia, which constrained the ability to detect effects of social media.

To our knowledge, the only previous investigation of longitudinal associations between social media use and paranoia is the one conducted by Berry et al [27]. While they did not directly assess the perception of web-based social company, they found that viewing social media newsfeeds predicted lower paranoia, whereas a perceived low social rank when using social media predicted higher paranoia. Their results, in line with community-based research on other aspects of well-being [59,60], indicate that social media can have both positive and negative associations with mental health depending on the contents of use. Our study measured social media use as a whole and did not distinguish among browsing, posting, or private messaging on social media. Hence, the results may be best understood as average correlates of media behaviors, spanning reductions in paranoia to increases therein, with differences in the direction of association that may arise from unmeasured individual, environmental, and media-related factors [61].

After probing a tentative (not statistically significant) interaction, we also found an association between listening to music and a subsequent decrease in paranoia in people with bipolar disorder. Potentially beneficial effects of music have been described in an internet-based sample of 457 adults with schizophrenia in the United States, where 42% of them reported that listening to music or audio files helped with managing auditory hallucinations [25]. The association in this study was modest in size, but it could be argued that in some individuals, listening to music promotes distraction from paranoid thoughts [17].

Differences in the likelihood of media use as a function of participant characteristics indicate the potential impacts of socioenvironmental adversity. Consistent with prior research [29,30], participants diagnosed with schizophrenia rather than bipolar disorder were less likely to report any versus no social media use and any versus no other forms of internet and computer use, a gap that may stem from lower socioeconomic status and functional impairments associated with schizophrenia. Black versus White participants and those who did not go to college were also less likely to report any versus no internet or computer use. Prior research from clinical samples in the United States and other western countries similarly show that lower educational attainment was associated with a lower likelihood of using digital media [26,32,62], and in a US sample of 322 people receiving psychiatric care, participants of racialized minority groups were less likely than White participants to use social media [29]. These associations may reflect the impact of systemic inequities on material deprivation and lower media-related literacy, which are some of the most common barriers to digital media use in clinical and nonclinical populations [31,32].

### Strengths and Limitations

This study is novel in its examination of media use in a large sample of participants with psychiatric diagnoses, who altogether contributed thousands of EMA surveys. By modeling associations between media use and paranoia at the within-person level, we were able to remove potential influences from time-invariant confounders such as sociodemographic features [63], thereby lending greater credence to possible causal effects of media use on paranoia. However, this method is not exempt from other sources of bias, including those from time-varying confounders: for example, a person’s use of social media could be triggered by a momentary increase in the availability of social support, which could be driving subsequent improvements in paranoia, thus acting as a time-varying confounder of the association between social media use and decreased paranoia. The validity of the media use questionnaire could not be assessed, but previous research has found moderate correlations between self-reported digital media use and app- or device-based activity logs [64]. The types of media evaluated here were not exhaustive and they aggregated heterogeneous media contents that may be differentially associated with paranoia. Each type of media was assessed with a single item, which was open to the respondent’s interpretation: for example, social media may have been understood by some participants and not others as including private messaging. Dichotomous assessments of media use did not provide information on the duration and contents of use (eg, browsing newsfeeds or messaging individuals). Paranoia EMA was evaluated with a single item, which had a significant, moderate correlation with clinician-rated trait paranoia but has not been validated in external samples. Our findings were also bound by a specific time frame of 3 assessments per day that produced intervals of hours between measures, potentially underestimating the frequency of media use. Effects of media use on paranoia may unfold over longer intervals than those assessed here (eg, after weeks of intense exposure). Lastly, while lower incidence of paranoia in the bipolar group might have constrained statistical power, the results raise the possibility of diagnosis-specific associations with media use, which may help better tailor media-based interventions.

Future research that provides more detailed assessments of media use over various time frames will be needed to examine these questions. Inclusion of putative mechanisms (eg, decreased loneliness) and concurrent symptoms (eg, negative symptoms) will also help elucidate the pathways between media use and paranoia. Another direction for future research is to consider associations of media use with paranoia in additional populations, notably adolescents and individuals with other mental health conditions such as borderline personality disorder and depression.

### Conclusions

This study found that social media use was associated with modest decreases in paranoia in a sample of individuals with schizophrenia or bipolar disorder. A better understanding of the social and cognitive possibilities of media technologies may help guide lifestyle counseling and media-based interventions for this population. However, systemic inequities and individual factors may hamper the accessibility of media, and the mental health correlates of media use may further vary as a function of specific contents and contexts of use.

## Supplementary material

10.2196/59198 Multimedia Appendix 1 Additional information.

## ABBREVIATIONS

*DSM-5*: *Diagnostic and Statistical Manual of Mental Disorders* (Fifth Edition)
EMA: ecological momentary assessment
PANSS: Positive and Negative Syndrome Scale
RMSSD: root-mean-square of successive differences
