# Supplementary material for: Media Use and Its Associations With Paranoia in Schizophrenia and Bipolar Disorder: Ecological Momentary Assessment
Source: JMIR Ment Health. 2024 Jul 3;11:e59198. doi: 10.2196/59198 (PMC11238023; doi:10.2196/59198)
Supplement: Multimedia Appendix 1 [file mental-v11-e59198-s001.docx]

**SUPPLEMENTARY MATERIAL**

Table of Contents

[Table S1. Characteristics of participants included in and excluded from analyses 2](#_Toc167299757)

[Figure S1. Paranoia as a function of lagged media use (sensitivity analysis with the full dataset) 3](#_Toc167299758)

[Figure S2. Media use as a function of lagged paranoia (sensitivity analysis with the full dataset) 4](#_Toc167299759)

[Figure S3. Paranoia as a function of lagged media use (sensitivity analysis adjusted for autocorrelation) 5](#_Toc167299760)

[Figure S4. Media use as a function of lagged paranoia (sensitivity analysis adjusted for autocorrelation) 6](#_Toc167299761)

[Figure S5. Paranoia as a function of concurrent media use 7](#_Toc167299762)

[Figure S6. Media use as a function of concurrent paranoia 8](#_Toc167299763)

# Table S1. Characteristics of participants included in and excluded from analyses

|  | **Included**  ***n=409*** | **Excluded**  ***n=37*** | **Effect size** |
| --- | --- | --- | --- |
| Site of recruitment, N (%): |  |  | **0.177** |
| The University of Texas at Dallas | 173 (42.3%) | 7 (18.9%) |  |
| University of Miami | 115 (28.1%) | 21 (56.8%) |  |
| University of California San Diego | 121 (26.6%) | 9 (24.3%) |  |
| Clinical group, N (%) |  |  | 0.073 |
| Schizophrenia | 189 (46.2%) | 22 (59.5%) |  |
| Bipolar disorder | 220 (53.8%) | 15 (40.5%) |  |
| Age in years, Median [25th;75th] | 40 [31;50] | 45 [35;49] | 0.038 |
| Gender, N (%): |  |  | 0.019 |
| Male | 162 (39.6%) | 16 (43.2%) |  |
| Female | 245 (59.9%) | 21 (56.8%) |  |
| Other | <5 (<1%) | 0 (0%) |  |
| Racialized group, N (%): |  |  | 0.077 |
| White | 212 (51.8%) | 21 (56.8%) |  |
| Black or African American | 138 (33.7%) | 11 (29.7%) |  |
| Asian | 20 (4.89%) | 0 (0%) |  |
| Other | 39 (9.54%) | 5 (13.5%) |  |
| Ethnicity, N (%): |  |  | **0.109** |
| Hispanic | 96 (23.5%) | 15 (40.5%) |  |
| Non-Hispanic | 312 (76.5%) | 22 (59.5%) |  |
| Educational attainment, N (%): |  |  | **0.135** |
| High school diploma or less | 123 (30.1%) | 19 (51.4%) |  |
| Some college | 148 (36.2%) | 12 (32.4%) |  |
| College degree or higher | 138 (33.7%) | 6 (16.2%) |  |
| Relationship status, N (%): |  |  | 0.037 |
| Not in a relationship | 216 (52.8%) | 22 (59.5%) |  |
| In a relationship | 193 (47.2%) | 15 (40.5%) |  |
| Positive symptoms, Median [25th;75th] | 13 [9;17] | 14 [11;19] | 0.044 |
| Reduced emotional experience, Median [25th;75th] | 5 [3;7] | 5 [3;8] | 0.017 |
| Reduced emotional expression, Median [25th;75th] | 4 [4;7] | 4 [4;10] | 0.048 |
| Depressive symptoms, Median [25th;75th] | 10 [0;18] | 10 [0;15] | 0.006 |
| Mania-related symptoms, Median [25th;75th] | 0 [0;3] | 0 [0;7] | 0.053 |

Effect sizes are Cramer’s V for categorical variables and Spearman correlations for continuous variables. Effect sizes ≥ 0.100 (in bold) are considered significant. Positive symptoms were measured with the positive symptom subscale of the Positive and Negative Syndrome Scale (PANSS); range: 7-49. Reduced emotional experience (range 3-21) and reduced emotional expression (range 4-28) were measured with negative symptom and general psychopathology items of the PANSS. Depressive symptoms were measured with the Montgomery-Åsberg Depression Rating Scale; range 0-60. Mania-related symptoms were measured with the Young Mania Rating Scale; range: 0-60.

# Figure S1. Paranoia as a function of lagged media use (sensitivity analysis with the full dataset)

Gamma mixed models with observations nested in individuals. In this sensitivity analysis, we did not exclude participants who completed less than one third of assessments. Predictors included mean levels of media use (for between-person associations) and lagged mean-centered media use (for within-person associations). All models include random slopes of mean-centered media use and random intercepts.

# Figure S2. Media use as a function of lagged paranoia (sensitivity analysis with the full dataset)

Generalized logistic mixed models with observations nested in individuals. In this sensitivity analysis, we did not exclude participants who completed less than one third of assessments. Predictors include mean levels of paranoia (for between-person associations) and lagged mean-centered paranoia (for within-person associations). The social media and TV model include a random slope of mean-centered paranoia. Models of music, reading, and other internet use include random intercepts only.

# Figure S3. Paranoia as a function of lagged media use (sensitivity analysis adjusted for autocorrelation)

Gamma mixed models with observations nested in individuals. In this sensitivity analysis, we adjusted for lagged paranoia. Predictors included mean levels of media use (for between-person associations) and lagged mean-centered media use (for within-person associations). All models include random slopes of mean-centered media use and random intercepts.

# Figure S4. Media use as a function of lagged paranoia (sensitivity analysis adjusted for autocorrelation)

Generalized logistic mixed models with observations nested in individuals. In this sensitivity analysis, we adjusted for lagged media use. Predictors include mean levels of paranoia (for between-person associations) and lagged mean-centered paranoia (for within-person associations). The social media and TV model include a random slope of mean-centered paranoia. Models of music, reading, and other internet use include random intercepts only.

# Figure S5. Paranoia as a function of concurrent media use

Gamma mixed models with observations nested in individuals. Predictors included mean levels of media use (for between-person associations) and concurrent mean-centered media use (for within-person associations). Models of social media, TV, reading and other internet use include random slopes of mean-centered media use. The music model only includes a random intercept.

# Figure S6. Media use as a function of concurrent paranoia

Generalized logistic mixed models with observations nested in individuals. Predictors include mean levels of paranoia (for between-person associations) and concurrent mean-centered paranoia (for within-person associations). The social media model includes a random slope of mean-centered paranoia. Models of TV, music, reading, and other internet use include random intercepts only.
